# Treatment discontinuation of remotely delivered cognitive remediation for schizophrenia: a systematic review and meta-analysis

**DOI:** 10.3389/fpsyt.2025.1643496

**Published:** 2025-10-30

**Authors:** Min Wen, Jie Zhang, Keqing Jiang, Juan Liu, Xiaodan Zhu

**Affiliations:** 1 School of Nursing, Ningxia Medical University, Yinchuan, China; 2 Department of Nursing, Shaanxi Rehabilitation Hospital, Xi’an, Shanxi, China; 3 Office of Hospital Director, General Hospital of Ningxia Medical University, Yinchuan, China

**Keywords:** schizophrenia, remote, treatment discontinuation, cognitive remediation, randomized controlled trial

## Abstract

**Introduction:**

Cognitive remediation therapy (CRT) is a pivotal treatment for cognitive impairments in patients with schizophrenia. However, there is a large proportion of community-dwelling patients with schizophrenia, and access to this therapy is not fully equalized across regions. The introduction of remotely delivered CRT presents a promising solution to these limitations. Given the substantial variation in settings for remotely delivered CRT, its treatment discontinuation and the factors influencing it remain to be fully elucidated. This meta-analysis aims to examine the treatment discontinuation of remotely delivered CRT and the factors influencing its treatment discontinuation.

**Methods:**

This study systematically searched PubMed, Embase, EBSCO, WHO ICTRP, ClinicalTrials, ProQuest, and BASE databases to identify randomized controlled trials involving remotely delivered CRT. Meta-analyses were performed using both random-effects and fixed-effects models. Subgroup and meta-regression analyses were employed to investigate potential factors affecting the treatment discontinuation of remotely delivered CRT.

**Result:**

The literature search yielded 2173 studies. 20 studies met the inclusion criteria and reported on 20 randomized controlled trials comparing remotely delivered CRT with control groups. Dropout rates were 22.96% for the remotely delivered CRT group and 20.82% for the control group. Meta-analysis results indicated no significant difference in dropout rate between the two groups (OR 0.99 [95% CI 0.78-1.25], *p*=0.901). Subgroup and meta-regression analyses identified that the development of cognitive strategies, facilitating the transfer of cognitive gains to everyday functioning, and the inclusion of all core CRT components were associated with lower rates of treatment discontinuation in remotely delivered CRT.

**Conclusion:**

Remotely delivered CRT demonstrates efficacy comparable to other forms of cognitive remediation, yet it exhibits a higher rate of treatment discontinuation. Future studies should consider the specificities of the target population and their environmental context, designing more meticulous and rigorous protocols to optimize the efficacy and treatment continuation of remotely delivered CRT.

**Systematic Review Registration:**

https://www.crd.york.ac.uk/prospero/, identifier CRD42024610531.

## 1 Introduction

Cognitive impairment ranks among the most pivotal symptoms of schizophrenia (1). This impairment precedes the initial episode of psychosis by nearly a decade and persists throughout the illness (2, 3). Antipsychotic medications are the primary treatment for schizophrenia (4, 5). However, their effectiveness is mainly limited to psychotic symptoms and offers limited efficacy in addressing cognitive impairment (6, 7). Consequently, the treatment of cognitive impairment in schizophrenia remains an unmet clinical need. Cognitive Remediation Experts Workshop defined cognitive remediation therapy (CRT) as an intervention targeting cognitive function, using scientific principles of learning with the goal of improving functional outcomes. Its effectiveness is enhanced when provided in a context (formal or informal) that provides support and opportunity for extending everyday functioning. The implementation of CRT comprises 4 key ingredients: the practice of cognitive exercises, attention to the development of cognitive strategies, an active trained therapist, and procedures to facilitate transfer of cognitive gains to everyday functioning (8). Several studies have substantiated that CRT is a significant method for facilitating functional recovery in individuals with schizophrenia (9–11).

Although CRT presents opportunities for cognitive and functional recovery in schizophrenia, and is endorsed by the Royal Australian and New Zealand College of Psychiatrists (RANZCP) advocating for its use (12). The National Institute for Health and Care Excellence (NICE) guidelines have yet to incorporate CRT into routine clinical practice (13). This may be attributed to the intrinsic characteristics of schizophrenia, which contribute to ongoing skepticism within the mental health field regarding the implementation of this therapy. Meta-analyses indicate that the treatment discontinuation rate for CRT ranges from approximately 13.7% to 16.58% (9, 14–16). Comparatively, the average treatment discontinuation rate for psychotherapy stands at 14% (16), suggesting a comparable level of treatment discontinuation for CRT.

It is worth noting that CRT is predominantly confined to specialized treatment facilities in major cities, with limited availability in developing countries or for out-of-hospital patients. This disparity results in unequal access to CRT for schizophrenia patients across different regions (17–19). Furthermore, the widespread outbreak of COVID-19 in 2019 further underscores the challenges associated with current mental health rehabilitation treatments (20).

Advances in interactive software development and healthcare delivery present a unique opportunity to overcome these limitations. A 2016 study noted that 81.4% of individuals with schizophrenia possess a cell phone (21), indicating that remote medicine could provide new avenues for CRT for people with mental illness and offer new options to address the current imbalance in resource allocation for implementing CRT (22). Additionally, a study confirmed that over 80% of current schizophrenia patients own telecommunication devices (21). Zhu (23), Medalia (24), Krzystanek (25) demonstrated that remotely delivered CRT is as effective as face-to-face treatment.

Moreover, after patients with schizophrenia are stabilized through inpatient treatment, most return to their families and communities, where they frequently require remotely delivered rehabilitation therapy (26). Compared to face-to-face interventions, remotely delivered CRT imposes greater demands on patient compliance (27, 28). It remains uncertain whether the treatment discontinuation of these two approaches is comparable. Furthermore, existing guidelines primarily recommend specific treatments for given situations but lack detailed guidance on factors influencing the treatment discontinuation of these treatments. To date, no meta-analyses have specifically addressed the treatment discontinuation of remotely delivered CRT in patients with schizophrenia. Consequently, this study systematically reviews the current evidence on the treatment discontinuation of remotely delivered CRT in schizophrenia and evaluates which characteristics may influence the treatment discontinuation of this treatment, with the aim of informing clinical practice.

## 2 Methods

A literature search for this study was conducted in accordance with the PRISMA guidelines (29) and was based on a protocol registered prospectively on PROSPERO (CRD42024610531). The search covered the period from 2000-01–01 to 2025-5–28 and utilized the PubMed, Embase, and EBSCO databases. Additionally, to minimize publication bias, a comprehensive grey literature search was performed, which included the WHO ICTRP, ClinicalTrials, ProQuest, and BASE. The search terms were (“schizophrenia” OR “psychosis”) AND ((“cognitive” OR “cognit*”) AND (“training” OR “remediation” OR “rehabilitation” OR “enhancement”)) AND (“computer” OR “phone” OR “tablet” OR “mobile” OR “internet” OR “online” OR “web” OR “app” OR “virtual” OR “telehealth” OR “remote”).

### 2.1 Inclusion and exclusion criteria

Eligible studies included: 1) those with at least 70% of participants diagnosed with schizophrenia; 2) randomized controlled trials comparing the efficacy of remotely delivered CRT with any other control condition (CRT could be used either as a stand-alone therapy or in combination with other interventions); 3) CRT administered remotely.

Exclusion criteria included: 1) literature not published in English; 2) studies involving face-to-face interventions combined with remote interventions.

### 2.2 Study selection

Screening was conducted by two independent reviewers, with any disagreements resolved by a third reviewer. Two independent reviewers assessed the validity of included studies using the risk-of-bias assessment tool from the Cochrane Collaboration (30). Studies were rated as having low, high, or unclear risk of bias. Any disagreements were resolved through discussion with the third reviewer.

### 2.3 Outcomes

The primary outcome was treatment discontinuation rate. In this meta-analysis, treatment discontinuation rate is defined as the proportion of patients who cannot adhere to continuous treatment for any reason during the 3–24 weeks of treatment (31, 32). This study aligns with this recommendation by measuring the treatment discontinuation of remotely delivered CRT using the odds ratio (OR) of the number of patients who discontinued treatment from the trial (OR = (treatment discontinuation from CRT/completers of CRT). Additionally, for studies with multiple treatment groups, only comparisons between remotely delivered CRT and control groups were considered.

### 2.4 Data analysis

All meta-analyses were performed using R 4.3.3. Statistical heterogeneity was evaluated through forest plots, the Q-test, and the *I*² statistic. Funnel plots and Egger’s test were employed to assess publication bias. If *p ≤* 0.1 and *I*²≥50% indicated significant heterogeneity among the studies, a random effects model was employed for the meta-analysis; if *p*>0.1 and *I*²<50% suggested no significant heterogeneity, a fixed effects model was utilized. In cases where heterogeneity was present between studies (*I*²>50%), subgroup analysis and sensitivity analysis were conducted to identify the sources of heterogeneity and to assess the robustness of the meta-analysis results. Funnel plots and Egger’s test were applied to evaluate publication bias, with a *p* value of less than 0.05 in Egger’s test indicating the presence of publication bias. Risk of bias was assessed using Review Manager 5.3. Descriptive statistics and analyses were conducted using SPSS 26.0.

Subgroup and meta-regression analyses were employed to explore potential influences on the treatment discontinuation of remotely delivered CRT. Dichotomous variables were represented by odds ratios (OR) and 95% confidence intervals (CI). For continuous variables, coefficients and 95% confidence intervals (CI) were employed. The list of moderating variables for the study is presented in **Table 1**. All analyses were considered significant at a *P* value <0.05.

**Table 1:** Effect of moderators.

Moderators	Heterogeneity			Treatment discontinuation rate		
	Study	*I^2^*(%)	*p*	Coefficient/OR (CI)	*Z/χ* ^2^	*p*
Publication year	20	97	<0.001	0.011(-0.012, 0.034)	0.857	0.355
Age(year)	20	0	0.999	0.001(-0.009, 0.010)	0.117	0.907
Education(year)	12	0	0.962	0.015(-0.162, 0.192)	0.029	0.865
Duration of illness (years)	9	0	0.992	0.006(-0.019, 0.030)	0.453	0.650
Age of onset (years)	6	0	0.910	-0.156(-0.043, 0.012)	-1.089	0.276
IQ	11	0	0.991	0.000(-0.015, 0.014)	-0.038	0.970
Baseline therapy dose (CPZeq)	9	0	0.968	0.000(-0.001, 0.001)	0.037	0.970
PANSS score	13	0	0.994	-0.006(-0.020, 0.009)	-0.765	0.444
Cognitive score	6	0	0.903	-0.028(-0.320, 0.263)	-0.190	0.849
Duration (weeks)	20	98	<0.001	0.005(-0.005, 0.015)	0.973	0.324
Intensity (season/week)	20	97	<0.001	0.026(-0.013, 0.065)	1.740	0.187
Intensity (hours/week)	18	98	<0.001	0.008(-0.043, 0.058)	0.088	0.767
Gender	7					
Female	7	48	0.14	0.119(0.014, 0.563)		
Male	7	12	0.01	0.064(0.014, 0.248)		
					9.90	0.624
payment	20					
Yes	10	37	0.112	0.284(0.252, 0.318)		
No	10	72	<0.001	0.185(0.099, 0.319)		
					2.16	0.142
Design	20					
Single-center trial	8	71	0.001	0.299(0.233, 0.375)		
Multi-center trial	12	50	0.023	0.276(0.245, 0.309)		
					0.44	0.507
Comparison category	20					
TAU	5	41	0.150	0.173(0.111, 0.258)		
Active TAU	3	68	0.045	0.348(0.168, 0.584)		
Nonspecific active control	12	51	0.020	0.280(0.249, 0.313)		
					5.70	0.058
Active and trained therapist (Element 1)	20					
Present	15	60	0.002	0.254(0.101, 0.329)		
Absent	5	64	0.025	0.225(0.139, 0.343)		
					0.020	0.651
Repeated practice of cognitive exercises (Element 2)	20					
Present	14	62	0.001	0.258(0.196, 0.332)		
Absent	6	57	0.041	0.216(0.130, 0.337)		
					0.42	0.517
Development of cognitive strategies (Element 3)	20					
Present	7	63	0.012	0.187(0.117, 0.286)		
Absent	13	49	0.025	0.299(0.267, 0.334)		
					4.88	**0.036**
Facilitate transfer of cognitive gains to everyday functioning (Element 4)	20					
Present	6	44	0.112	0.165(0.109, 0.242)		
Absent	14	46	0.030	0.303(0.271, 0.337)		
					9.45	**0.002**
Interventions including all core elements (1,2,3,4)	20					
Present	4	39	0.177	0.180(0.124, 0.254)		
Absent	16	56	0.004	0.285(0.238, 0.338)		
					5.47	**0.019**

Note: Bold values indicate statistically significant differences at P < 0.05.

Additionally, to address the occurrence of zero event counts in the meta-analysis, a continuity correction was applied by replacing zero with 0.1. This method helped to avoid mathematical issues in the calculation of effect sizes and ensures the stability and accuracy of the results.

## 3 Results

### 3.1 Included studies

**Figure 1:**
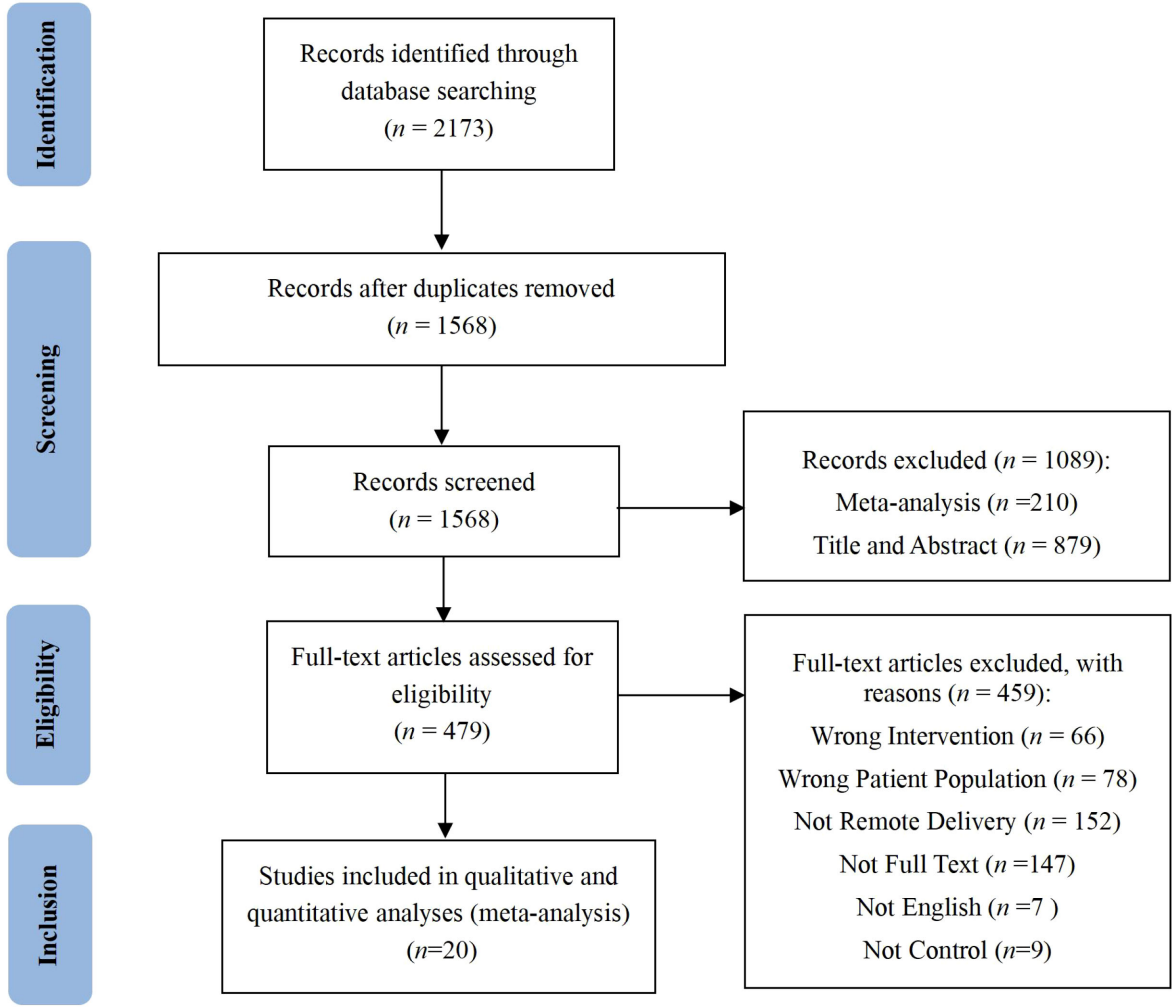
PRISMA flow diagram.

The study selection process is illustrated in **Figure 1**. Our search yielded 2173 records, of which 20 randomized controlled trials were included in the meta-analysis. These studies provided 20 independent comparisons between remotely delivered CRTs and a control group, involving a total of 1977 participants.

The 20 included studies were published between 2012 and 2023 (25, 33–51). Of these studies, one study lasted 3 weeks (5.00%), nine lasted 8 weeks (45.00%), six lasted 10–13 weeks (30.00%), and four lasted 24 weeks (20.00%). Remotely delivered CRT was compared to treatment as usual (TAU) in 25.00% of studies, active TAU in 15.00%, and nonspecific active control in 60.00% (see **Supplementary Data**). 8 studies were single-center trials (40.00%), while the remaining 12 were multicenter trials (60.00%) see **Table 2**.

**Table 2:** Descriptive characteristics of included studies.

Study	Group	Baseline/ post-intervention	Dropout rate(%)	Females(%)	Duration (weeks)	Comparison category
Biagianti (2017) (33)	CRT	41/26	36.59	26.92	8	Nonspecific active control
	TAU	33/21	36.36	9.52	8	
Cassetta (2016)* (34)	CRT	54/54	0.00	\	10	TAU
	TAU	27/27	0.00	\	10	
Cella (2023) (35)	CRT	24/18	25.00	50.00	8	TAU
	TAU	24/19	20.83	37.50	8	
Donohoe (2018) (36)	CRT	48/25	47.92	35.42	8	Active TAU
	TAU	42/30	28.57	42.24	8	
Fisher (2015) (37)	CRT	63/43	31.75	27.91	8	Nonspecific active control
	TAU	58/43	25.86	23.26	8	
Garety (2015) (38)	CRT	51/47	7.84	\	3	Nonspecific active control
	TAU	50/43	14.00	\	3	
Hargreaves (2015) (39)	CRT	30/22	26.67	22.73	8	Active TAU
	TAU	26/26	0.00	30.77	8	
Ikezawa (2012) (40)	CRT	62/51	17.74	39.22	24	TAU
	TAU	21/21	0.00	36.36	24	
Iwata (2017) (41)	CRT	29/28	3.45	75.86	12	TAU
	TAU	31/28	9.68	74.19	12	
Krzystanek (2019) (25)	CRT	199/142	28.64	42.71	24	Nonspecific active control
	TAU	91/60	34.07	34.07	24	
Krzystanek (2020) (42)	CRT	199/142	28.64	42.71	24	Nonspecific active control
	TAU	91/60	34.07	34.07	24	
Loewy (2022) (43)	CRT	81/56	30.86	23.75	8	Nonspecific active control
	TAU	66/48	27.27	27.69	8	
Montemagni (2021) (44)	CRT	33/23	30.30	21.74	24	Nonspecific active control
	TAU	33/25	24.24	60.00	24	
Nahum (2021) (45)	CRT	76/55	27.63	30.26	10	Nonspecific active control
	TAU	71/53	25.35	30.99	10	
Panizzutti (2019) (46)	CRT	47/47	0.00	31.91	10	Nonspecific active control
	TAU	43/43	0.00	18.61	10	
Ramsay (2018) (47)	CRT	22/22	0.00	36.36	8	Nonspecific active control
	TAU	22/22	0.00	27.27	8	
Ramsay (2021) (48)	CRT	22/21	4.55	38.10	8	Nonspecific active control
	TAU	22/22	0.00	27.27	8	
Saleem (2014) (49)	CRT	5/5	0.00	40.00	8	TAU
	TAU	6/6	0.00	16.67	8	
Souto (2018) (50)	CRT	30/30	0.00	20.00	12	Active TAU
	TAU	31/30	3.23	23.33	12	
Van (2021) (51)	CRT	34/29	14.71	26.47	13	Nonspecific active control
	TAU	39/28	28.21	30/77	13	

Drop out is defined as the premature withdrawal of participants from a clinical trial due to any reason resulting in non-completion of the full study protocol. (Dropout rate = incomplete patients/patients at baseline.

*For multi-arm trials, intervention groups were combined and compared against the shared control group to avoid double-counting, as per Cochrane guidelines.

### 3.2 Treatment discontinuation of remotely delivered cognitive remediation therapy

The overall dropout rate was 22.96% for remotely delivered CRT and 20.82% for the control groups (25.83% and 23.63%, respectively, when the no-withdrawal trial was excluded). The proportions of dropping out for any reason in the two groups are shown in **Table 3**. Among them, treatment discontinuation was the most common reason for attrition in both the remotely delivered CRT group (62.12%).

**Table 3:** Proportion of different reasons of dropout.

Reasons for dropout	Group	No.	Proportion (%)
Lost to follow-up	TAU	52	30.23
	CRT	83	3144
Withdrawal	TAU	101	58.72
	CRT	164	62.12
Other reason	TAU	19	11.05
	CRT	17	6.44
Technical problems	TAU	8	4.65
	CRT	12	4.55
Missed assessment	TAU	11	6.40
	CRT	5	1.89

Drop out is defined as the premature withdrawal of participants from a clinical trial due to any reason resulting in non-completion of the full study protocol. Lost to follow-up is defined as a situation in which participants fail to attend scheduled visits, resulting in the investigators’ inability to obtain their final outcome data. Withdrawal refers to the termination of the control treatment for TAU group or CRT treatment for CRT group that is initiated by the researcher or by the participants themselves.

**Figure 2:**
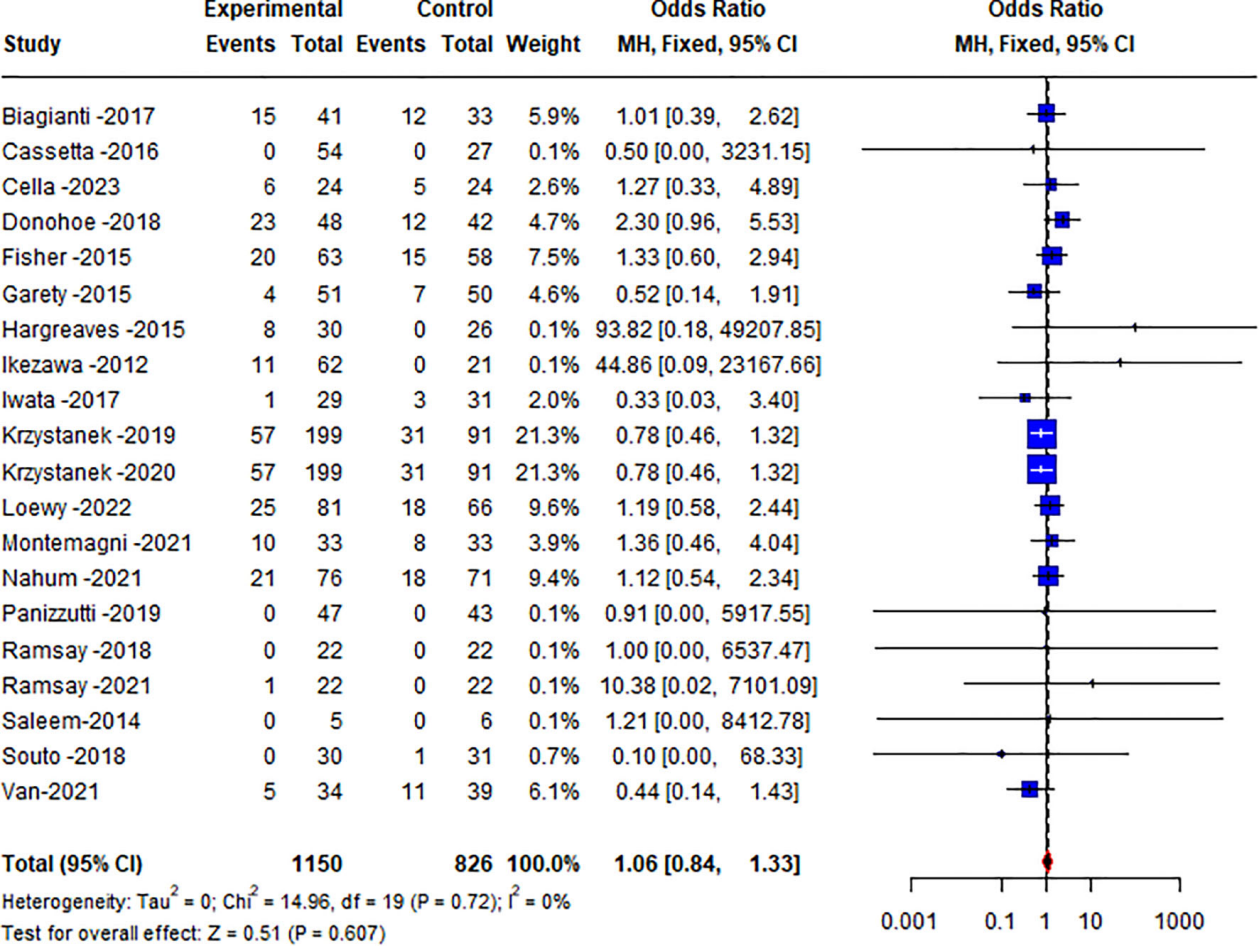
Forest plot of treatment discontinuation of remotely delivered CRT.

Overall heterogeneity was not statistically significant (Q = 14.96, df = 19, p=0.720, *I*² = 0%). In the meta-analysis, there was no significant difference between the remotely delivered CRT treatment and control groups in the dropout (OR 1.06 [95% CI 0.84-1.33], p=0.607) (see **Figure 2**). Sensitivity analysis using a random effects model yielded similar results (OR 0.99 [95% CI 0.78-1.25], p=0.901). The funnel plot indicated a possible absence of publication bias (*t*=1.28, *p*=0.215, see **Supplementary Figure 1**). Risks of bias are summarized in **Supplementary Figure 2**.

### 3.3 Moderator effects

The publication year, age, years of education, duration of illness, age of onset, IQ, PANSS score, baseline cognitive score, duration of training, intensity, gender, payment, study design, and comparison category had no significant effect on the treatment discontinuation.

In subgroup analyses conducted to explore the effects of the core components of remotely delivered CRT on treatment discontinuation, the presence of an active and trained therapist (OR 0.254 [95% CI 0.101-0.329] vs. OR 0.225 [95% CI 0.139-0.343], *χ*² =0.020, *p*=0.651) and repeated practice of cognitive exercises (OR 0.258 [95% CI 0.196-0.332] vs. OR 0.216 [95% CI 0.130-0.337], *χ*² = 0.42, *p*=0.517) did not have a significant effect. In contrast, the development of cognitive strategies (OR 0.187 [95% CI 0.117-0.286] vs. OR 0.299 [95% CI 0.267-0.334], *χ*² = 4.88, *p* =0.036) and facilitating the transfer of cognitive gains to everyday functioning (OR 0.165 [95% CI 0.109-0.242] vs. OR 0.303 [95% CI 0.271-0.337], *χ*² = 9.45, *p*=0.002) were associated with lower treatment discontinuation. Additionally, studies that included the core components of CRT demonstrated lower treatment discontinuation compared to those missing core elements (OR 0.180 [95% CI 0.124-0.254] vs. OR 0.285 [95% CI 0.238-0.338], *χ*² = 5.47, *p*=0.019), see **Table 3**.

## 4 Discussion

This study represents the first systematic and comprehensive assessment of the dropout rate of remotely delivered CRT for patients with schizophrenia. The overall dropout rate was 22.96% for the remotely delivered CRT and 20.82% for the control group, with no statistically significant difference observed between the two groups.

However, this study exhibited a higher dropout rate compared to the rates reported in Vita’s meta-analysis (15), which were 16.58% for CRT and 15.21% for control groups. Several factors may account for this discrepancy. First, the relatively small number of studies included in this analysis could have compromised the stability and representativeness of the statistical outcomes. Second, Vita’s meta-analysis encompassed multiple forms of CRT and included both inpatient and outpatient patients, whereas the present analysis focused solely on outpatients receiving remotely delivered CRT (15). It is well-recognized that remotely delivered CRT poses specific challenges, such as the need for technical support and the absence of direct support and supervision from healthcare professionals, which likely contributed to the higher treatment discontinuation observed among outpatients compared to those in hospital settings. Nonetheless, it is noteworthy that there was no significant difference in the dropout rate between outpatients in the remotely delivered CRT and the control group in this meta-analysis. Thus, remotely delivered CRT can still be considered an effective alternative treatment option in contexts where medical resources are scarce or patient mobility is limited. Future studies may incorporate individualization in standard intervention protocols and design flexibility-time interventions to minimize patient treatment discontinuation rates. At the same time, patients’ families could be encouraged to participate in a supervisory role, and patients may also perceive higher levels of social support and compliance with the intervention.

Contrary to previous studies (14, 15, 52) where IQ and years of education are significant predictors of CRT efficacy on cognitive and functional outcomes (53–55), this study did not observe a significant effect of lower IQ and fewer years of education on the treatment discontinuation of remotely delivered CRT. The reason for this analysis may lie in the enhanced version of CRT therapy utilized, which is more straightforward and accessible in terms of operation and training. This optimization could render remotely delivered CRT adaptable to patients with varying IQ and educational backgrounds, thereby improving the therapy’s treatment continuation. Consequently, the influence of IQ and education on treatment discontinuation may be diminished or counteracted. Additionally, the limited number of studies included in this analysis compared to other meta-analyses might have contributed to the lack of significant differences in the findings. This highlights the individual variability among people with schizophrenia and their differing capacities to process information and undergo training.

Additionally, this study further investigated the impact of the four core components of CRT (presence of an active and trained therapist, repetitive practice of cognitive exercises, development of cognitive strategies, and facilitating the transfer of cognitive gains to everyday functioning) on the treatment discontinuation of remotely delivered CRT (9, 56, 57). Established guidelines and meta-analyses have verified the substantial influence of these components on treatment outcomes. Contrary to the conventional belief that complexity in treatment leads to higher treatment discontinuation, this study observed that the development of cognitive strategies, facilitate transfer of cognitive gains to everyday functioning, and inclusion of these four components were associated with lower treatment discontinuation. These divergent results may be due to the presence of certain components that help participants better apply the outcomes of cognitive training to real-life situations. This practical application provides positive feedback that can significantly enhance patients’ motivation and attitude towards continuing treatment.

However, akin to the findings from Vita (15), this study also noted that the presence of an active and trained therapist, and the repetitive practice of cognitive exercises did not significantly affect treatment discontinuation. This may be because these elements alone do not directly influence patients’ motivation to engage in therapy, but may indirectly reduce treatment discontinuation rates by supporting the development of the other two core components (15).

The findings demonstrate that remotely delivered CRT exhibits comparable dropout rates to in-person delivery, supporting its value in expanding treatment adherence—particularly in remotely delivered cognitive remediation. To enhance treatment adherence, interventions should focus on strengthening cognitive strategy training and real-world application rather than reducing program complexity. Furthermore, as patients exhibit variability in their capacity to process information, implementing personalized approaches that accommodate different cognitive profiles and difficulty levels is recommended to optimize outcomes.

## 5 Limitation

This study also presents several limitations. Firstly, the meta-analysis was restricted to studies published in English, which may have introduced selection bias. Although this limitation is not methodologically addressable, it could restrict the applicability of the study results to the treatment discontinuation across different income countries. Secondly, many included studies did not comprehensively report the specific reasons for participant withdrawal, thereby limiting the more in-depth understanding and analysis of the factors influencing access and participation. Thirdly, among the 20 included studies, 3 (15%) did not report gender distribution data, which may compromise the completeness of gender-difference analyses and introduce potential bias. Fourth, given inconsistent definitions of the treatment discontinuation time frame in existing studies (31, 32, 58) and considering the intervention periods analyzed herein, the treatment discontinuation timeframe was set at 8 to 24 weeks in this study. Consequently, the results here only represent interventions lasting 3 to 24 weeks. Finally, treatment discontinuation rates likely capture to some extent participant perceptions and motivation for CRT, but treatment discontinuation can occur for reasons unrelated to acceptability. There are many other reasons related to treatment discontinuation (e.g., someone may very much like the CRT but may still elect to drop out of the study due to moving, increase in responsibilities, increased stressors, etc.).

## 6 Conclusion

Although the results of this meta-analysis differ from those of previous studies, they should not be viewed as contradictory. The discrepancies are likely due to the substantial differences in the environments of face-to-face versus remotely delivered CRT, as well as between in-hospital and out-of-hospital settings. These results suggest the need for a more nuanced understanding and analysis of the various factors that affect the treatment discontinuation of remotely delivered CRT. Future studies could conduct large-scale international studies and aggregate data to achieve a more comprehensive grasp of the key variables influencing motivation and participation. Additionally, the intricate interactions among these factors and their specific manifestations across different patient treatment modalities and populations warrant further exploration. This would help optimize the design and implementation of remotely delivered CRT, enhancing its overall treatment continuation and effectiveness.

## Data Availability

The original contributions presented in the study are included in the article/**Supplementary Material**. Further inquiries can be directed to the corresponding authors.
