# Correction: Treatment discontinuation of remotely delivered cognitive remediation for schizophrenia: a systematic review and meta-analysis

**DOI:** 10.3389/fpsyt.2025.1744074

**Published:** 2025-11-19

**Authors:** Min Wen, Jie Zhang, Keqing Jiang, Juan Liu, Xiaodan Zhu

**Affiliations:** 1 School of Nursing, Ningxia Medical University, Yinchuan, China; 2 Department of Nursing, Shaanxi Rehabilitation Hospital, Xi’an, Shanxi, China; 3 Office of Hospital Director, General Hospital of Ningxia Medical University, Yinchuan, China

**Keywords:** schizophrenia, remote, treatment discontinuation, cognitive remediation, randomized controlled trial

The funder “Key R&D Projects of Ningxia (No.: 2019BFG02023)” was erroneously omitted.

The original version of this article has been updated.

