# Supplementary material for: Treatment discontinuation of remotely delivered cognitive remediation for schizophrenia: a systematic review and meta-analysis
Source: Front Psychiatry. 2025 Oct 30;16:1643496. doi: 10.3389/fpsyt.2025.1643496 (PMC12612859; doi:10.3389/fpsyt.2025.1643496)
Supplement: Supplementary file 1 [file Supplementaryfile1.docx]

Supplementary Material

# Supplementary Data

Classifying comparison conditions were specifically categorized into three groups: 1. Treatment as usual (TAU: drug treatment/case management only, waiting lists, insufficient details); 2. Active TAU (including multidisciplinary rehabilitative programs); 3. Nonspecific active control (controlling for non-specific aspects and matched with CR for duration and schedule, e.g. social stimulation, leisure activities, computer activities).

# Supplementary Figures and Tables

For more information on Supplementary Material and for details on the different file types accepted, please see [here](https://www.frontiersin.org/guidelines/author-guidelines#supplementary-material).

## Supplementary Figures


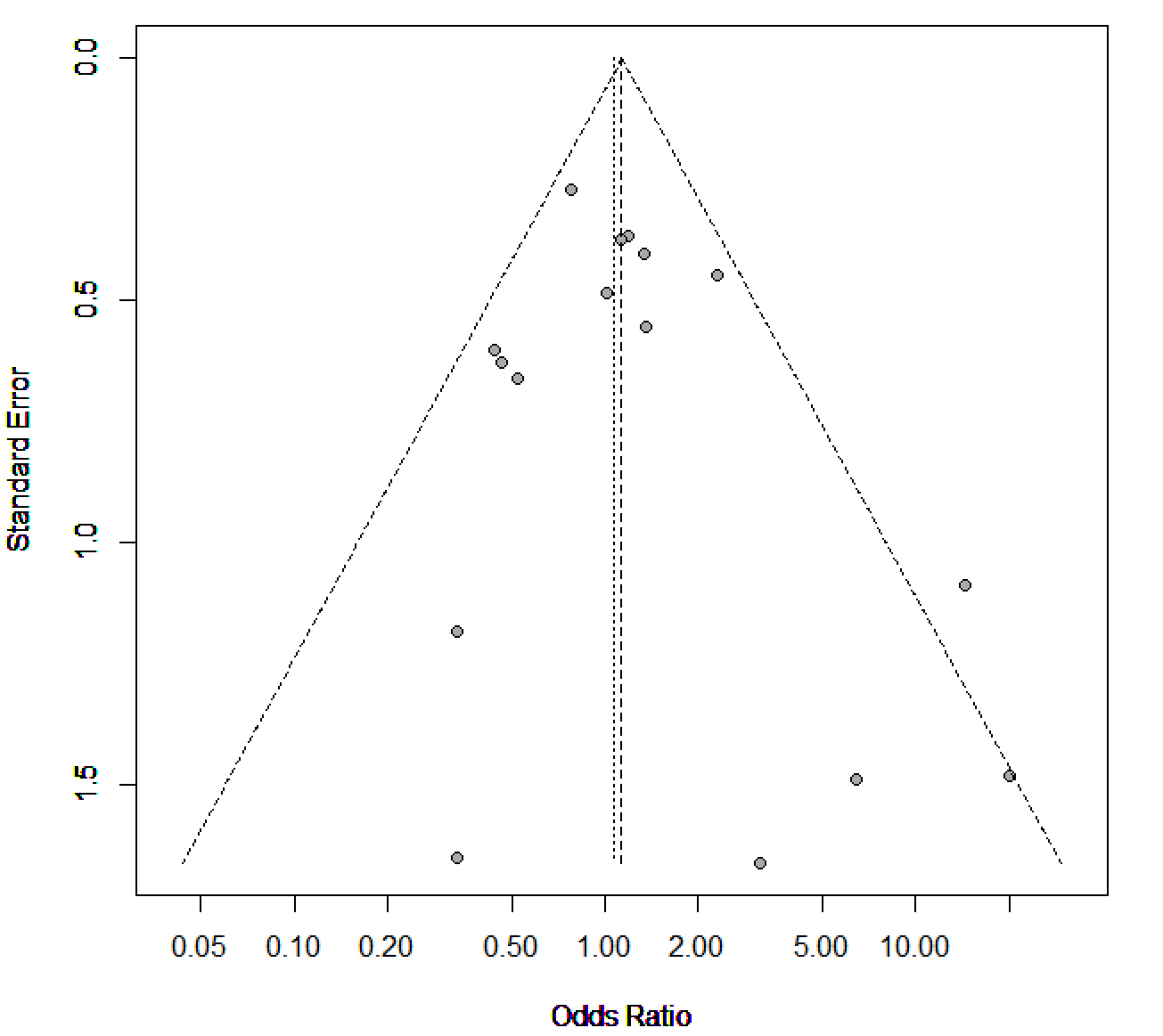


**Supplementary Figure 1.** The funnel plot of remotely delivered CRT.


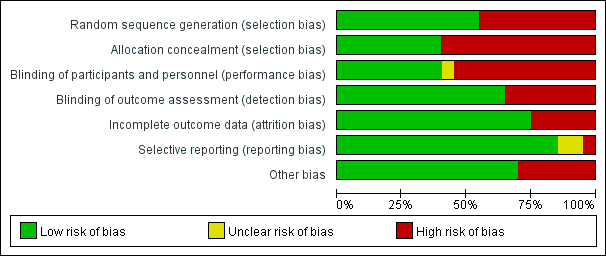


**Supplementary Figure 2.a** Risk of bias summary (presented as a graph).

**
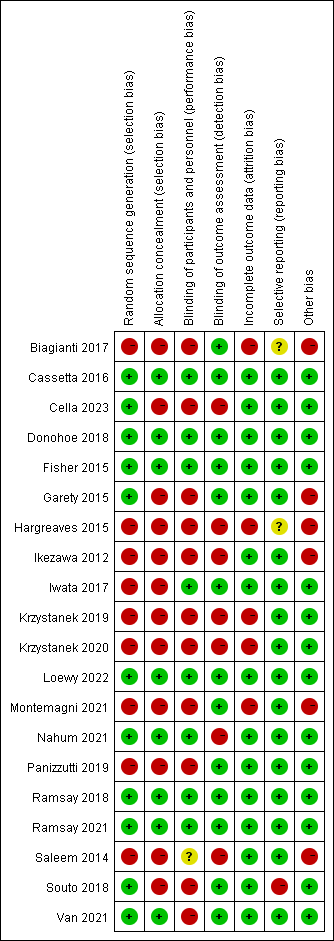
Supplementary Figure 2.b** Risk of bias summary (presented as a table).

**Note:** (+) Low risk of bias; (?) Unclear risk of bias; (-) High risk of bias
